# Incomplete Penetrance and Variable Expressivity: From Clinical Studies to Population Cohorts

**DOI:** 10.3389/fgene.2022.920390

**Published:** 2022-07-25

**Authors:** Rebecca Kingdom, Caroline F. Wright

**Affiliations:** Institute of Biomedical and Clinical Science, Royal Devon & Exeter Hospital, University of Exeter Medical School, Exeter, United Kingdom

**Keywords:** penetrance, expressivity, variant intepretation, genomic sequencing, rare disease

## Abstract

The same genetic variant found in different individuals can cause a range of diverse phenotypes, from no discernible clinical phenotype to severe disease, even among related individuals. Such variants can be said to display incomplete penetrance, a binary phenomenon where the genotype either causes the expected clinical phenotype or it does not, or they can be said to display variable expressivity, in which the same genotype can cause a wide range of clinical symptoms across a spectrum. Both incomplete penetrance and variable expressivity are thought to be caused by a range of factors, including common variants, variants in regulatory regions, epigenetics, environmental factors, and lifestyle. Many thousands of genetic variants have been identified as the cause of monogenic disorders, mostly determined through small clinical studies, and thus, the penetrance and expressivity of these variants may be overestimated when compared to their effect on the general population. With the wealth of population cohort data currently available, the penetrance and expressivity of such genetic variants can be investigated across a much wider contingent, potentially helping to reclassify variants that were previously thought to be completely penetrant. Research into the penetrance and expressivity of such genetic variants is important for clinical classification, both for determining causative mechanisms of disease in the affected population and for providing accurate risk information through genetic counseling. A genotype-based definition of the causes of rare diseases incorporating information from population cohorts and clinical studies is critical for our understanding of incomplete penetrance and variable expressivity. This review examines our current knowledge of the penetrance and expressivity of genetic variants in rare disease and across populations, as well as looking into the potential causes of the variation seen, including genetic modifiers, mosaicism, and polygenic factors, among others. We also considered the challenges that come with investigating penetrance and expressivity.

## Introduction

Approximately 72% (Nguengang Wakap et al., 2020) of all rare diseases are genetic in origin, and most of these are thought to be monogenic in nature (Haendel et al., 2020). Rare, deleterious variants are known to cause thousands of different genetic disorders in humans (Boycott et al., 2017; Rahit and Tarailo-Graovac, 2020), and while the molecular basis of over 6,000 monogenic diseases has been uncovered (OMIM, 2022), with more than 200,000 pathogenic variants described (QIAGEN, 2022; Stenson et al., 2017), the genetic basis of most rare disorders remains to be determined. With advances in next-generation sequencing (NGS) and the increasing availability of whole exome/genome sequencing (WES/WGS), the study of genotype–phenotype relationships has become more widespread as determining how the genotype causes a phenotype is a fundamental step toward understanding disease pathology (Stephanou et al., 2019). Protein-coding variants that are associated with disease phenotypes directly link DNA variation to altered protein function or dosage and to the phenotypic outcome, and so much of what we know about the genotype–phenotype relationship is based on the study of rare variants that cause monogenic disease (Chong et al., 2015). Monogenic genotypes can be highly predictive for specific individual disorders, but sometimes this relationship can be complicated, with some damaging dominant monogenic variants not following the expected Mendelian inheritance patterns (Schacherer, 2016). Individuals with the same genotype can display distinctly different clinical phenotypes (McDermott et al., 2017; Kumar et al., 2019; Crawford et al., 2021), including being clinically asymptomatic. Currently, there are gaps in translating how the individual genomic variation affects phenotypic presentation and how genetic variants exert their functional impact to cause disease.

The study of genetic disease has often been divided into rare monogenic forms of disease and more common polygenic complex disorders (Claussnitzer et al., 2020). Current evidence suggests that these groups may be more overlapping than previously thought as the genetic variation present across the genome highlights the complexity underlying the phenotypic presentation. There are both rare variants in individual genes that cause monogenic forms of complex disease (Vuckovic et al., 2020; Muse et al., 2021) and common variants that affect the severity of monogenic disease (Niemi et al., 2018; Goodrich et al., 2021). Such complexity makes investigating the genotype–phenotype relationships more complicated, which is only exacerbated by erroneous variant associations due to study design problems (Wright et al., 2019a). Human genetic diversity displays considerable variability, with individual genomes differing from the reference at 4.1–5 million sites (Auton et al., 2015). Although most variation is common and predicted to be functionally neutral (Ng et al., 2008), each individual has on average 85 heterozygous and 35 homozygous protein-truncating variants (PTVs) (Lek et al., 2016). Population cohort studies have shown that the average genome contains around 200 very rare variants per person (Gudmundsson et al., 2021) and 54 variants previously reported as disease-causing, including 7.6 rare non-synonymous coding variants in monogenic disease genes (Lek et al., 2016; Walsh et al., 2017). Variant interpretation is an ongoing challenge within diagnostic medicine, making understanding the phenotypic consequences of underlying genetic variation a key aim of genomics research.

### Incomplete Penetrance and Variable Expressivity

**FIGURE 1:**
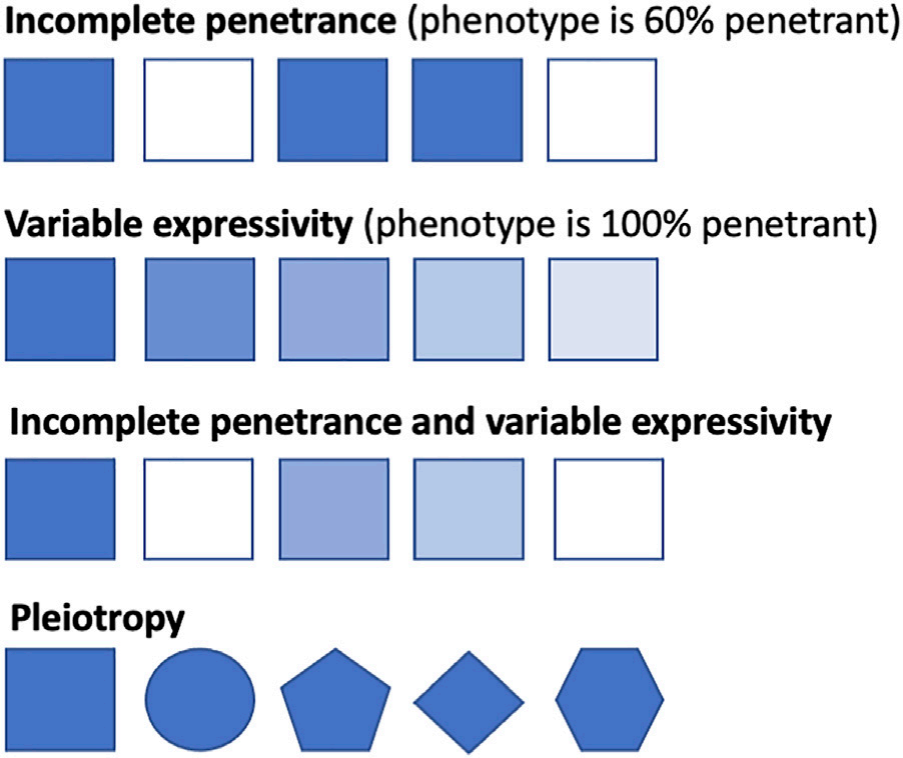
Conceptual representation of penetrance, expressivity, and pleiotropy. Squares represent individuals with the same genotype, with shaded squares indicating the individual displays the related phenotype and non-shaded squares indicating the individual does not display the related disease phenotype. Line one shows incomplete penetrance, where 60% of the individuals display the related phenotype. Line two shows that all individuals display the related phenotype, from severe manifestations to milder presentations. Line three shows incomplete penetrance and variable expressivity, where the genotype varies both in the severity of presentation and in penetrance across the population. Line four shows pleiotropy, whereby different phenotypes are caused by variants (represented by different shapes) in one gene.

A deleterious genotype should be no more prevalent in the population than the disease that it causes (Minikel et al., 2016). However, the same genetic variant can result in different disease presentations in different people, from clinically asymptomatic to severely affected, even among members of the same family (Mahat et al., 2021). The proportion of individuals who possess a particular genotype and exhibit the expected clinical symptoms is defined as the penetrance of that genotype (Cooper et al., 2013; Shawky, 2014). If everyone with the genotype presents with clinical symptoms by a particular age, then it is said to be fully penetrant, whereas if it falls below this, it is said to exhibit reduced or incomplete penetrance. Genotypes can also display variable expressivity, where the severity of the phenotype caused by the genotype can vary among affected individuals (Shawky, 2014) (Table 1); this differs from pleiotropy, where different variants in the same gene can cause different, potentially unrelated phenotypes that may even be categorized as different diseases (Ittisoponpisan et al., 2017) (Figure 1). Although penetrance, expressivity, and pleiotropy are three distinct concepts, biological reality means that their overall effects often overlap, especially in population cohorts where it is difficult to identify the cause of the phenotypic diversity. Multiple distinct phenotypes, in aggregate, could either be classified as a single more severe phenotype or different disease subtypes. As these three are likely to be caused by overlapping or similar mechanisms (Gruber and Bogunovic, 2020), especially in genetically heterogenous conditions, we will discuss them together in this review.

**TABLE 1:** Examples of variable expressivity in monogenic diseases. Deleterious variants in these genes are known to cause a spectrum of phenotypes, from severe disease to mild subclinical effects.

Causal gene	Severe phenotype	Milder phenotype
*HOXD13*	Synpolydactyly (extra fused digits) (Ibrahim et al., 2016)	Short digits (Johnston et al., 2015; Zhang et al., 2020b)
*KCNQ4*	Deafness (Li et al., 2021a)	Mild hearing loss (Johnston et al., 2015)
*SGCE*	Myoclonus dystonia (Raymond et al., 1993)	Dystonia/Writer’s cramp (Gerrits et al., 2009; Johnston et al., 2015)
*KRT16*	Pachyonychia congenita (Smith et al., 1993)	Blistered feet (Johnston et al., 2015; Li et al., 2021b)
*FLCN*	Birt-Hogg-Dube syndrome (Schmidt and Linehan, 2018)	Mild fibrofolliculomas (Johnston et al., 2015)
*SFTPC*	Lung disease (Nathan et al., 20201983)	Abnormal lung diffusion capacity (Somaschini et al., 2005; Johnston et al., 2015)
*FBN1*	Severe Marfan syndrome (Díaz de Bustamante et al., 2012; Aubart et al., 2018)	Mild Marfan phenotypes (tall, thin, slender fingers) (Dietz et al., 1993)
*ERCC4*	Xeroderma pigmentosum (Kraemer et al., 1993)	Higher likelihood of sunburn (Wright et al., 2019a)
*FLG*	Ichthyosis vulgaris (Akiyama, 2010)	Eczema (Wright et al., 2019a)
*POLG*	Childhood onset Alpers-Huttenlocher syndrome (Kammenga, 2017)	Deterioration of eye muscles (Neeve et al., 2012)

**FIGURE 2:**
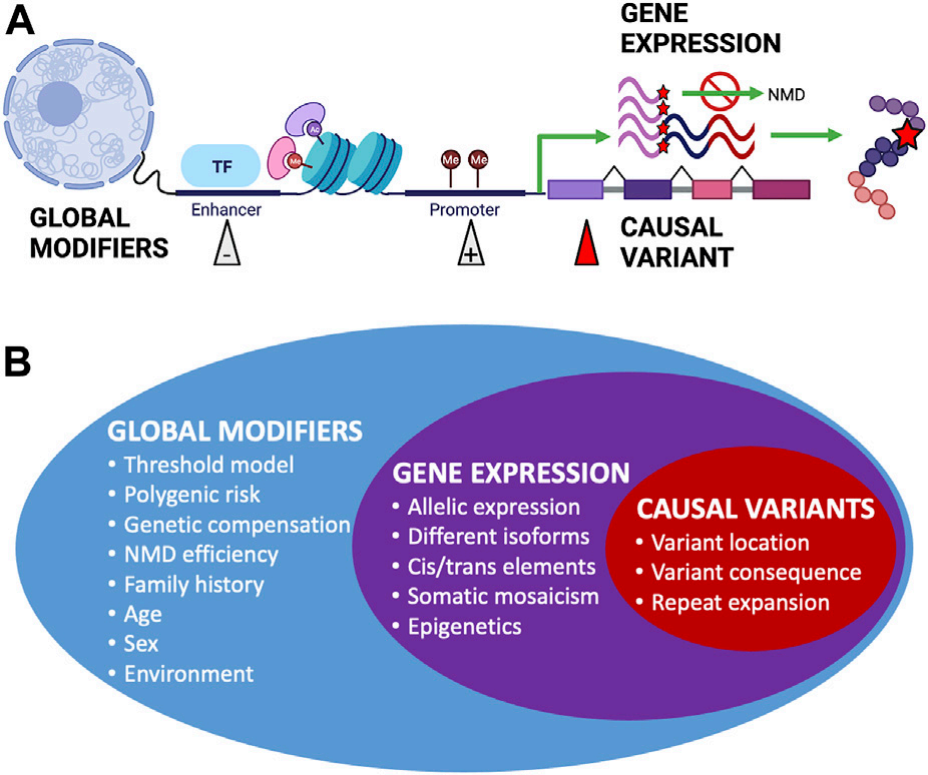
Factors affecting penetrance and expressivity. **(A)** Examples of different biological mechanisms that can affect the overall penetrance and expressivity of monogenic disease-causing genetic variants. Figure created using BioRender.com. **(B)** Summary of factors affecting penetrance and expressivity across the genome, from global modifiers that can have wide-ranging overall effects to expression of the gene containing causal variants and to specific causal variants that have more distinctive effects.

Incomplete penetrance can be observed in both dominant and recessive conditions. However, the cause of variability in genotype–phenotype correlations can be difficult to elucidate; phenotypic variation has been observed in mice with identical environmental and genetic backgrounds, including variability in lethality for gene knockouts despite the introduction of identical variants (Dickinson et al., 2016). Establishing that a identified variant is the sole (or primary) cause of an individual’s clinical phenotype can be difficult (Shieh, 2019), which is an important concern when it comes to diagnosis and providing accurate genetic counseling, and such difficulties can lead to incorrect or delayed diagnosis (Maroilley and Tarailo-Graovac, 2019). The widespread presence of incomplete penetrance and variable expressivity through many overlapping mechanisms (Figure 2) can explain why apparently unaffected parents can pass on pathogenic variants to affected offspring (McDermott et al., 2017) and why seemingly healthy individuals’ genomes can contain a large number of putatively damaging variants and yet not suffer any obvious adverse effects (Xue et al., 2012).

Although databases of clinically identified variants in affected individuals are useful for assessing pathogenicity (van Rooij et al., 2020), population-based datasets that include WES/WGS alongside phenotypic and medical information are increasingly important for investigating the penetrance and expressivity of these variants. Large population cohort studies have shown the occurrence of apparently pathogenic variants is much higher than previously estimated through small clinical or familial cohort studies (Wright et al., 2019a; Lacaze et al., 2020; van Rooij et al., 2020), and their frequency highlights either the incomplete penetrance, variable expressivity, or misclassification of such variants. The existence of PTVs in dosage-sensitive genes in healthy individuals also remains problematic when it comes to determining pathogenicity (Cummings et al., 2020). The potential for genomic technologies and WGS to detect individuals at risk of genetic disease is enormous, but incomplete penetrance and variable expressivity present a challenge for clinicians, especially when an incidental finding occurs without any prior clinical indication, leading to uncertainty over whether a clinical phenotype will develop, and if so, when. This problem is highlighted when testing unselected population cohorts, who may or may not have phenotypes of relevance to genomic findings at the point of testing. To understand how genetic disorders develop, we need to consider how deleterious variants interact with the rest of the variation in the genome and how variation can affect phenotypic presentation. This may also identify targets that help prevent disease progression (Downs et al., 2019). The presence of putatively pathogenic variants in asymptomatic adults also highlights the possibility that there are disease resistance mechanisms we can identify through the sequencing of general population cohorts.

### Clinical Versus Population Cohorts

**FIGURE 3:**
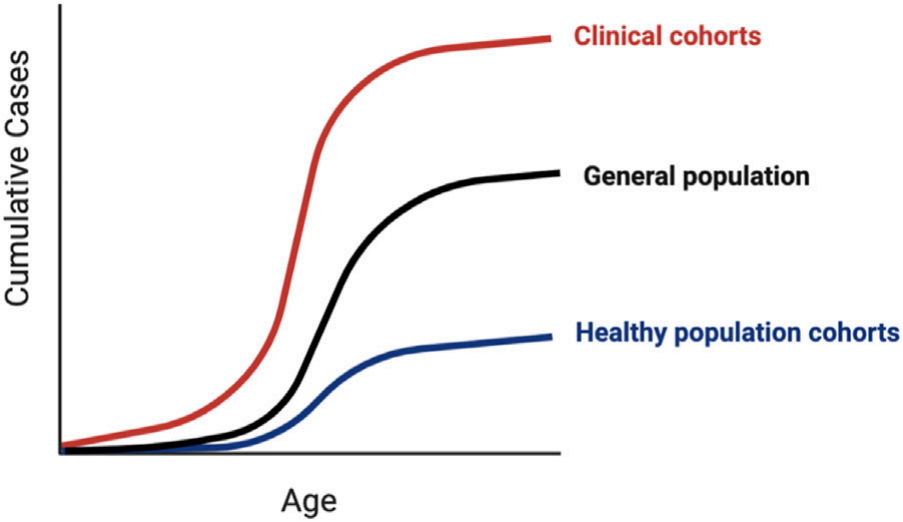
Penetrance in clinical versus population cohorts. Penetrance of genetic variants identified in clinical cohorts tends to be higher than the same variants identified in population cohorts, which can manifest as earlier disease onset, less severe disease, or a larger proportion of affected individuals. Due to inherent ascertainment biases in both types of cohorts, the penetrance of variants in the general unselected population is likely to lie somewhere in-between.

Traditionally, rare pathogenic variants were identified in small phenotypically enriched clinical cohorts of individuals and families with similar monogenic disease. Population cohorts allow us to utilize the information from small clinical studies to investigate the penetrance of variants in the general “healthy” population, where such severe monogenic phenotypes are likely to be depleted, and the potential to identify the causes of clinical heterogeneity. Ascertainment bias can occur with any study design, with volunteer population cohorts tending to be healthier than the average individual (Fry et al., 2017) and clinical cohorts tending to have more severe phenotypes. Estimates of the maximum and minimum variant effect sizes across different ascertainment contexts are needed to avoid falsely predicting that a significant proportion of the healthy population is at risk for a monogenic condition (Flannick et al., 2013). The proportion of individuals affected and the average age of onset (i.e., age-dependent penetrance) can vary depending on the ascertainment context (Figure 3). For example, individuals with putatively pathogenic variants in *HNF1A* and *HNF4A*, known for causing maturity-onset diabetes of the young (MODY), develop diabetes significantly later or not at all when tested outside of the context of clinical referrals for the suspected MODY (Mirshahi et al., 2021).

For almost all human genetic diseases, individual variability in the phenotype is influenced by background variation in the genome. As genetic testing has become more widely available, both through healthcare systems, direct-to-consumer testing (Stoeklé et al., 2016), our understanding of how genomic variation affects disease progression and prevalence has become significantly more important, both for clinical utility (Shieh, 2019) and for our functional understanding of the disease (Tarailo-Graovac et al., 2017). Variation in the genome can predispose individuals to disease through traditional monogenic variants that disrupt physiological pathways and exert a large effect on the phenotype, or through the accumulation of polygenic effects that involve many variants of small effect sizes in different pathways (Fahed et al., 2020), or as is increasingly becoming clear, through their combined effect.

Within population cohorts, penetrance estimates for monogenic variant carriers average 60% or lower for most conditions (Goodrich et al., 2021), illustrating that many individuals have highly penetrant, pathogenic variants in known monogenic disease-causing genes who never develop the corresponding phenotype (Chen et al., 2016). Generally, 70% of the “Wellderly” healthy aging cohort, all of whom reached 80 without any chronic diseases, had one heterozygous deleterious variant in genes listed in the American College of Medical Genetics and Genomics (ACMG) secondary findings (Erikson et al., 2016). Similarly, one in 75 (1.3%) of healthy elderly individuals in the APSREE trial carried a previously identified pathogenic variant, including in Lynch syndrome and familial hypercholesterolemia genes, without having the associated phenotype (Lacaze et al., 2020). These cases demonstrate that carrying such pathogenic variants does not always cause the associated disease and that other mechanisms may contribute to the protection of human health, including genetic modifiers that ‘rescue’ individuals from a disease phenotype.

## Causal Variants

### Variant Location and Consequence

For genetically heterogenous monogenic diseases, the penetrance and expressivity can vary between different genes or variants, with the same phenotype potentially caused by numerous different variants across multiple genes (Wright et al., 2018). Even within the same gene, some deleterious variants in known monogenic disease genes may exhibit complete penetrance, while others show incomplete or low penetrance. Variation can be due to functional redundancy of genes, or the location and type of variant, with missense and PTVs in the same gene often causing different phenotypes. For example, hereditary angioedema can show great phenotypic diversity, even among members of the same family, and individuals with missense variants in *SERPING1* typically display a milder and later onset of disease than patients with PTVs (Speletas et al., 2015). In contrast, missense variants in *BMPR2* cause earlier and more severe pulmonary hypertension than PTVs in the same gene (Austin et al., 2009).

Pathogenic PTVs typically cause disease through loss of function (LoF) due to degradation of the RNA by nonsense-mediated decay (NMD) (Lu and Krebber, 2021). NMD is an mRNA surveillance pathway that recognizes and degrades damaged mRNA transcripts that would produce misfolded or shortened proteins that can accumulate in the cell and initiate the endoplasmic reticulum (ER) stress response (Haeri and Knox, 2012). However, the production of a variant protein can either exacerbate disease severity through the accumulation of toxic proteins in the cell (Nguyen et al., 2014) or alleviate it through providing a residual function that protects against haploinsufficiency-mediated disease in the heterozygous state (van Leeuwen et al., 2017; Coban-Akdemir et al., 2018; Kennedy et al., 2019), meaning the occurrence of NMD can affect phenotypic severity depending on the mechanism of disease. PTVs may also cause LoF through aberrant splicing (Cummings et al., 2020), which is also regulated by NMD (Lareau and Brenner, 2015). In some cases, the location of NMD boundaries at the 5′ and 3′ ends of genes containing causal variants can explain phenotypic variation between individuals with different PTVs in the same gene (Nagy and Maquat, 1998; Lindeboom et al., 2016). For example, PTVs located outside of the region that triggers NMD in *SOX10* escape NMD and produce proteins that have dominant-negative activity, causing the severe complex neurological disorder PCWH, whereas PTVs located within the NMD region produce transcripts that are recognized by NMD and removed, causing the relatively milder WS4 syndrome *via* haploinsufficiency (Inoue et al., 2004; Miller and Pearce, 2014). This variability in penetrance or expressivity could potentially be classed as distinct subtypes of disease, with different variants causing disease through different mechanisms and producing distinct syndromes. Pathogenic variants in *KAT6B* show a similar disease manifestation, with two distinct syndromes depending on whether NMD is triggered or not (Zhang et al., 2020a). Variants in *KAT6A* cause severe intellectual disability (ID) and neurodevelopmental disorders (NDD), with late PTVs more likely to cause a severe phenotype, compared to 60% of early PTVs which conferred a mild phenotype (Kennedy et al., 2019), potentially due to whether NMD is activated or not. The position of the PTV within the gene has also been seen to modulate the severity of clinical phenotypes in Marfan syndrome (Taniguchi et al., 2021) and Charcot-Marie-Tooth disease (Pipis et al., 2022). Disease due to *SFTPB* variants typically presents in neonates as respiratory distress syndrome, resulting in death within the first few months; variants that allow partial production of the SP-B protein confer longer survival times and later onset of disease, whereas the variants that cause complete deficiency of SP-B due to NMD cause fatal neonatal respiratory distress syndrome (van Moorsel et al., 2021).

Missense variants can also result in LoF due to substantially reduced protein function or stability (Høie et al., 2022). Although many missense variants have little or no effect, they can result in conformational changes, increased protein misfolding, and aberrant protein trafficking, which can lead to intracellular retention or accumulation, increased ER stress, activation of the unfolded protein response, or increased pro-apoptotic signaling and apoptosis (van Moorsel et al., 2021). Some missense variants, small insertions/deletions, and gene duplications can also result in gain of function (GoF) effects due to increased activity (Niday and Tzingounis, 2018), increased protein production (Stefl et al., 2013), or *via* protein products that gain a new damaging function (Li and Babu, 2018). Some GoF variants can exhibit a more severe phenotype than LoF variants in the same gene; for example, GoF variants in *KCNA2* were associated with more severe epilepsy phenotypes than LoF variants (Syrbe et al., 2015). Where in a gene a variant is located can affect the mechanism of disease, as well as penetrance and expressivity through molecular subregional effects (Platzer et al., 2017); the impact of a variant depends on whether it is located at sites that undergo post-translational modification, within sites that are critical for tertiary and quaternary structure, at protein–protein interaction interfaces or ligand binding sites, or inside versus outside of functional domains (Faure et al., 2022). For example, missense variants in *GRIN2A* located in transmembrane or linker domains were more frequently associated with severe developmental phenotypes than those located elsewhere, such as within amino-terminal or ligand-binding domains (Liu et al., 2021), with a wide range of phenotypes observed from normal to mild epilepsy, to severe developmental phenotypes and epileptic encephalopathy (Strehlow et al., 2019); similarly, GoF variants in highly conserved regions of the potassium channel of *KCNA2* were associated with more severe epileptic encephalopathy than variants located elsewhere (Masnada et al., 2017). An improved understanding of the protein structure and the functionality of interacting domains will help elucidate specific variant effects on the resulting phenotypic presentation (Ittisoponpisan et al., 2021).

Finally, there are a small but increasing number of pathogenic non-coding variants that have been identified as causes of monogenic diseases. These variants can operate either through LoF or GoF mechanisms by altering the gene or isoform expression (Ellingford et al., 2021). For example, biallelic variants in the *PTF1A* enhancer are a well-established cause of recessive pancreatic agenesis through tissue-specific LoF (Weedon et al., 2014); *de novo* LoF variants in the 5′ untranslated region (UTR) of *MEF2C* have been shown to account for around a quarter of developmental disorder diagnoses in this gene (Wright et al., 2021); and a single GoF variant that creates a novel promoter has been shown to cause α-thalassemia (Bozhilov et al., 2021). However, establishing the pathogenicity of non-coding variants is often much more challenging than coding variants, and thus, studies of penetrance and expressivity of these variants are likely to lag behind.

### Size of Repeat Expansions

Repeat expansion disorders are caused by genomic expansions of short tandem repeat (STR) sequences that either affect the gene expression or protein sequence (Paulson, 2018), with the penetrance and expressivity affected by the number of repeats (Table 2). Anticipation is often observed in families due to molecular instability around the repeats; in each generation, the repeat length can increase, resulting in the earlier onset of disease and increased severity. For example, Fragile X syndrome is caused by the expansion of over 200 repeats in the CGG motif in the 5′UTR of *FMR1* on the X chromosome, resulting in hypermethylation of the promoter, silencing the gene (Hagerman et al., 2017). Fragile X exhibits incomplete penetrance and reduced expressivity, with 100% of males and 60% of females presenting with ID and 50–60% of males and 20% of females diagnosed with autism spectrum disorder (ASD) (Payán-Gómez et al., 2021). Wild type (WT) alleles contain <44 CGG repeats, while full mutations in affected individuals typically have >200 repeats. Those with premutation alleles of 55–200 repeats have milder phenotypes than full mutation carriers, although they have an increased risk of Fragile X-associated tremor/ataxia syndrome (Cabal-Herrera et al., 2020) and primary ovarian insufficiency prior to age 40 (Fink et al., 2018) compared to WT. Monotonic dystrophy shows a similar mechanism, with unaffected individuals having 5–37 CTG repeats in the 3′UTR of *DMPK* and fully affected individuals having >80 repeats (although repeats of >1,000 have been seen in congenitally affected children (Morales et al., 2016)), with an number of repeats correlating with the earlier age of onset.

**TABLE 2:** Trinucleotide repeat disorders with varying penetrance depending on the number of repeats present.

Disease	Gene	STR	Non-penetrant	Intermediate penetrance	Full penetrance
Spinocerebellar ataxia 8	*ATXN8OS*/*ATXN8* (Perez et al., 2021)	CTG/CAG	<91	92–106	>107
Spinal muscular atrophy	*SNM1* (Laskaratos et al., 2021)	CAG	<34	35–46	>47
Fragile X	*FMR1* (Hagerman et al., 2017)	CGG	<44	45–200	>200
Huntington’s	*HTT* (Kay et al., 2016)	CAG	<36	37–39	>40
ALS	*C9orf72* (DeJesus-Hernandez et al., 2011)	GGGGCC	<23	24+	>700
Friedrich’s Ataxia	*FXN* (Kim et al., 2011)	GAA	<34	35–99	>100

Although the number of repeats accounts for a large proportion of variable expressivity, there are still missing genetic factors accounting for differences in the age of onset. For example, in Huntington’s disease, a lower number of N-terminal CAG repeats in *HTT* is associated with reduction in penetrance and later onset of clinical symptoms (Kay et al., 2016), but while the number of repeats is inversely correlated with the age of onset of motor symptoms, they only account for 70% of the variability (Holmans et al., 2017). The remaining unexplained variance displays a high degree of heritability, suggesting further genetic modifiers (Arning, 2016). Additional genetic variants in the DNA mismatch repair pathway have been linked with anticipation and overall severity of disease, and functional studies showing the knockout of base-excision repair or transcription-coupled repair pathways in animal and cellular models of nucleotide repeat disorders can inhibit the expansion and reduce the phenotypic severity (Goula and Merienne, 2013; Massey and Jones, 2018). Variants in the DNA repair gene *MSH3* have also been linked with differences in disease severity through somatic instability (Flower et al., 2019). As non-penetrant individuals will not necessarily come to clinical attention and large triplet repeats are hard to genotype accurately using NGS (Bahlo et al., 2018), it is suspected that individuals with fewer than 41 CAG repeats in *HTT* may exist at a higher frequency than previously expected in the general asymptomatic population (Kay et al., 2016).

## Gene Expression

### Variation in Allelic Expression

It has been hypothesized that the differential expression of alternative alleles in the gene containing causal variants could affect the presentation of phenotypic traits in individuals with identical genotypes. This mechanism has been proposed primarily for dominantly inherited conditions where haploinsufficiency is the cause of the disease (Ahluwalia et al., 2009; Jordan et al., 2019), including Lynch syndrome (Hesson et al., 2015) and hypertrophic cardiomyopathy (HCM) (Glazier et al., 2019), where an allelic imbalance could cause either higher expression of the WT allele, thus compensating for the haploinsufficiency and resulting in reduced penetrance, or lower expression of the WT allele, thus exacerbating the haploinsufficiency and resulting in higher penetrance. Significant allelic imbalance has been observed in up to 88% of genes in human tissues, potentially caused by genetic modifiers or stochastic factors (Aguet et al., 2017), and has been identified as both tissue-specific and genome-wide in mouse models (Pinter et al., 2015). Structural variants such as duplications that are in *trans* with a pathogenic LoF variant can alleviate the potential clinical phenotype when disease would be caused by haploinsufficiency, by providing an additional WT copy of a gene, thus resulting in a normal level of gene expression (Servetti et al., 2021), as has been observed in DiGeorge syndrome (Carelle-Calmels et al., 2009). Additional variants in the untranslated regions of mRNA can also affect the translational efficiency and gene expression can also vary widely across tissues, highlighting the importance of sequencing disease-relevant tissue in the interpretation of genetic variation (Cummings et al., 2017; Mignone et al., 2002). Compared to synonymous variants, rare missense variants show a significant reduction in allelic expression across many tissues in proportion to their predicted pathogenicity, suggesting deleterious variants are depleted from highly expressed haplotypes (Castel et al., 2018). Some highly differentially expressed genes have been shown to contain fewer disease-associated variants (Chen et al., 2008), which are less likely to accumulate on haplotypes that are highly expressed, or in high-penetrance combinations (Castel et al., 2018). For example, genetically heterogenous monogenic eye disorders display both incomplete penetrance and variable expressivity and also display significant variability in gene expression levels throughout the population (Green et al., 2020). The differential expression of alleles has also been shown to play a role in the variable expressivity of Marfan’s syndrome (Aubart et al., 2015).

The differential expression of alleles can also potentially cause recessive conditions to present in a dominant fashion. For example, Zellweger spectrum disorder (ZSD) is an autosomal recessive disorder caused by deleterious variants in any of 13 PEX genes, with the most common cause being variants in *PEX1* or *PEX6*. Affected heterozygous carriers have been identified with ZSD despite lacking a second pathogenic allele, with all affected heterozygotes presenting with the allelic overexpression of the variant allele compared to WT, and a common polymorphism has been linked to this allelic overexpression (Falkenberg et al., 2017). In HCM, the proportion of sarcomeric proteins produced by variant alleles can vary with the allelic expression, and 30–80% of the sarcomere structure can be made up of proteins with reduced function (Marian and Braunwald, 2017; de Marvao et al., 2021), causing variation in overall phenotypic severity.

Stochastic variation within normal cellular and developmental processes can potentially be amplified by disease-causing variants and thus play a role in incomplete penetrance and variable expressivity (Binder et al., 2015). Random monoallelic expression (RME) is the transcription of only one allele from a homologous pair and can be constitutive, with all cells expressing the same allele throughout (as seen in imprinted genes), or somatic, with individual cells showing variation in expression levels (Eckersley-Maslin and Spector, 2014). Overall levels of RNA in cell populations tend to be stable, but dynamic allelic fluctuation through RME can present variability in the gene expression. Genes that show little RME are mostly housekeeping genes that have higher expression levels (Eckersley-Maslin and Spector, 2014). Although no variation in the disease trait has yet been definitively linked to somatic RME, conceptually it could explain the phenotypic variation either through alteration of gene dosage or the higher expression of a variant allele. RME during embryonic development has been tentatively linked with variation in developmental disorders such as Holt-Oram syndrome (Gui et al., 2017). Model organism research has suggested stochastic variation in the gene expression can affect the expressivity of variant genotypes, with 20% of genes causing variation in phenotypes in two different isolates with defined genetic backgrounds in *C. elegans* (Vu et al., 2015). Phenotypic variability has also been observed in inbred mice with a defined genetic background (Dickinson et al., 2016), as well as in monozygotic (MZ) twins (Baranzini et al., 2010), suggesting the influence of stochastic molecular events in variable expressivity.

### Variation in Isoform Expression

Production of different transcripts of genes may also lead to the differential expression of traits and explain why potentially deleterious variants in haploinsufficient genes are found in population cohorts. Annotations based on transcription levels of different isoforms in haploinsufficient genes identified that 23% of LoF variants are in under-expressed exons and had similar effect sizes to synonymous variants (Cummings et al., 2020). In monogenic cardiomyopathies caused by LoF variants in the giant muscle protein titin, studies of *TTN* expression levels indicate that LoF variants found in unaffected population cohorts occur predominantly in exons that are absent from the most highly expressed transcripts and thus do not cause the phenotypic effect associated with deleterious variants (Begay et al., 2015; Akinrinade et al., 2019). Similarly, haploinsufficiency of *TCF4* causes the highly penetrant Pitt-Hopkins syndrome (Kharbanda et al., 2016; Sirp et al., 2021), PTVs identified in these gene in unaffected individuals were all found to be located in minimally expressed exons (Aguet et al., 2017), suggesting that functional protein can be made in the presence of these variants. The expression of tissue-specific isoforms can also affect the penetrance of a genotype, potentially resulting in distinct disease subtypes. For example, *CACNA1C* has two clinically important isoforms with mutually exclusive exons that explain two different forms of Timothy syndrome; pathogenic variants across the widely expressed transcript produce a multi-system disorder (type 1), while pathogenic variants in the alternative exon of a transcript predominantly expressed in the heart are much rarer and result in more severe cardiac-specific defects and fewer syndromic phenotypes (type 2) (Dick et al., 2016). Further examples are likely to be uncovered through large-scale analysis of isoform expression in different tissues and at different times.

### *Cis*- and *Trans*-Acting Genetic Modifiers

Variants in regulatory regions can affect the phenotypic presentation of disease by altering the gene expression and through modulation of deleterious genetic variants found in associated protein-coding regions (Scacheri and Scacheri, 2015), potentially affecting the penetrance and expressivity of the monogenic variant. *Cis*-acting elements are DNA sequences located on the same haplotype as the gene they affect, whereas *trans*-acting factors are proteins or elements that bind to the *cis*-acting sequences to affect the gene expression. Variants in these non-coding regions can have multiple downstream effects, through interactions with other genetic features or through effects on monogenic variants (van der Lee et al., 2020). Small changes within transcription factor binding or expression can lead to dysregulation that affects multiple genes within the same regulatory network (van der Lee et al., 2020) and therefore could potentially alter the final phenotypic presentation. *Cis*-regulatory variants have been identified that modify the penetrance of coding variants and therefore contribute to disease risk or presentation. Pathogenic coding variants are depleted from higher-expressed haplotypes with *cis*-regulatory variants in the general population (Castel et al., 2018), suggesting that individuals who present with a disease phenotype may have an enrichment of *cis*-regulatory variants that increase the expression of the pathogenic allele, compared to individuals who are asymptomatic who have an enrichment of ‘protective’ regulatory variants that decrease the expression and, therefore, penetrance of the pathogenic allele (Castel et al., 2018).

Upstream open reading frames (uORFs) are tissue-specific *cis*-regulators of protein translation found in the 5′UTR region of protein-coding genes, and variants that alter uORFs can affect whether a deleterious protein-coding variant causes a disease phenotype or not and may alter the phenotypic presentation of the disease (Silva et al., 2019). Active translation of a uORF can reduce downstream protein levels by up to 80% *via* several mechanisms, including the production of a peptide that stalls the translating ribosome (Young et al., 2016) and termination at a uORF stop codon that can trigger NMD (Lee et al., 2021a). Variation that either introduces or removes uORF start or stop codons can, therefore, affect the phenotypic presentation, and uORF variants may also have a role in disease pathology (Whiffin et al., 2020). Variants in the downstream 3′UTR region may also play a role in regulation of the gene expression through altering the mRNA stability or translational efficiency (Jansen, 2001; Mignone et al., 2002; Steri et al., 2018). For example, a common single nucleotide polymorphism (SNP) downstream of *GATA6* has been shown to reduce its expression, potentially resulting in a more severe pancreatic agenesis phenotype when found in *trans* with a LoF variant in the same gene (Kishore et al., 2020). Similarly, polymorphisms in the 3′UTR region of *KCNQ1* have been suggested to alter the expression of the *cis* allele, either increasing the severity of the disease or reducing it through an uneven expression of WT or variant alleles (Amin et al., 2012). However, an attempt to replicate this in a diverse group of population cohorts found no association between the identified polymorphisms and the severity of disease (Kolder et al., 2015), highlighting the difficulties with trying to identify non-coding modifiers of rare disease, both in clinical cohorts and population studies.

Approximately 400,000 candidate enhancer regions have been identified in the human genome, with an average of around 20 enhancers per gene (The ENCODE Project Consortium, 2012; Yokoshi et al., 2020). Non-coding variants within enhancer regions can be a cause of phenotypic diversity through alterations in gene expression, therefore affecting overall disease phenotype presentation (Sun et al., 2018). Although identifying non-coding variants that affect disease presentation can be very difficult, there are some notable examples. A large study identified an SNP in an intronic enhancer of *RET* that appeared to increase the penetrance of Hirschsprung disease in patients with rare *RET*/coding variants (Emison et al., 2010). Intronic variants have also been suggested to affect the penetrance of coding variants in patients with Stargardt disease, where a deep intronic variant has been shown to be a major *cis*-acting modifier of the most common pathogenic variant in *ABCA4* (Zernant et al., 2018; Lee et al., 2021b). A small study also suggested that SNPs in promoter regions affect the severity of arrhythmias among individuals with LoF variants in *SCN5A* (Park et al., 2012). Variants that create novel binding sites for transcription factors have been implicated in affecting penetrance through altering the gene expression, including a common non-coding polymorphism that alters the hepatic expression of *SORT1* (Musunuru et al., 2010), contributing to myocardial infarction. Further WGS research is needed to identify non-coding variants that affect gene expression levels.

Genes are often associated with multiple *cis*-regulatory elements through topologically associated domains (TADs) (Delaneau et al., 2019). These domains are thought to affect the gene expression and mediate the effects of *cis-* and *trans-*regulatory factors through the 3D conformation of chromatin, and therefore, variants in these domains can affect penetrance and expressivity of genotypes (Galupa and Heard, 2017; McArthur and Capra, 2021). Although the expression of some genes has been shown to be unaffected by changes in TADs (Williamson et al., 2019), the creation of new TADs has been implicated in the pathogenicity of rare duplications (Franke et al., 2016). Alterations to the 3D chromatin structure within and between TADs can lead to mis-alignment of genes, enhancers, and silencers, affecting transcriptional control of the gene expression (Boltsis et al., 2021). Variants in TAD loops may have no effect on healthy individuals but could affect disease presentation in those with an underlying monogenic variant (Lu et al., 2020). Common genetic variants in *cis-*regulatory domains can affect the gene expression, and rare variants have been identified that disrupt the structure of the domain (Epstein, 2009; van der Lee et al., 2020), and both could contribute to varying phenotypic expressivity of identical protein-coding sequences by causing changes in upstream mechanisms of gene regulation. Structural changes that affect transcription factor binding can lead to functional gene expression changes (McArthur and Capra, 2021), as seen in the *EPHA4* locus, where deletions or duplications that overlap the TAD boundary can cause severe limb malformations (Lupiáñez et al., 2015), while deletion of the entire locus does not (Helmbacher et al., 2000), which is thought to be due to differential gene enhancer associations.

### Somatic Mosaicism

Postzygotic *de novo* mutations that occur during cell division can result in somatic genetic variation that differs between cells, leading to mosaicism (Biesecker and Spinner, 2013). Monogenic disease is usually less severe in mosaic individuals than those who have the same variant expressed constitutively and depending upon which cells or tissues contain the pathogenic variant, mosaicism can result in non-penetrance or reduced expressivity (Hervé et al., 2015). Somatic mosaicism is suspected to be more widespread than is usually detected, especially when testing only a single tissue sample that may or may not contain the clinically relevant variant(s), although NGS is making it easier to identify lower-level genetic changes (Domogala et al., 2021; Chen et al., 2022).

Mosaic somatic variants have been suggested to be more representative than germline variants of the true diversity and range of potential variation in human disease as genotypes that are lethal in the constitutive form can be identified when present as mosaic (Bickley et al., 2014; Alswied et al., 2021). These include variants that cause osteogenesis imperfecta, where a mosaic father presented with mild symptoms, but the constitutive form was incompatible with life (Wallis et al., 1990), Proteus syndrome (Cohen, 2014) and CLOVES syndrome (Ferreira et al., 2021), two overgrowth disorders that are lethal in the constitutive form, and various mosaic aneuploidies (Leon et al., 2011). Alternatively, mosaic individuals can display different or milder phenotypes than those with germline variants in the same gene. For example, mosaic individuals with a variant in *HRAS* present with benign keratinocytic epidermal nevi (“woolly hair”) (Honda et al., 2017), whereas those with the same constitutive variant have the more severe Costello syndrome (Gripp et al., 1993). Other diseases that have been demonstrated to show a milder phenotype when caused by somatic mosaicism include telangiectasis (Tørring et al., 2017) and polycystic kidney disease (Hopp et al., 2020). Mosaic genotypes can also display varying phenotypes that include segmental forms of the constitutive disease, such as segmental neurofibromatosis type 1, where clinical manifestations are only shown in certain parts of the body (Jindal et al., 2019). In addition to presenting with variable expressivity, mosaic variants can also be incompletely penetrant. In individuals with primary immunodeficiencies, 80% of mosaic individuals were clinically asymptomatic, with the remaining 20% exhibiting partial clinical symptoms (Mensa-Vilaró et al., 2019; Gruber and Bogunovic, 2020). Similarly, mosaic chromosomal aneuploidy has been shown to be incompletely penetrant in population cohorts, with women who had 45,X/46,XX mosaicism presenting with normal reproductive lifespan and birth-rate and no cardiovascular complications, compared to those with the non-mosaic genotype (Tuke et al., 2019). Unaffected parents with mosaic pathogenic variants can pass their genotype onto their offspring as a constitutive germline variant, so an incompletely penetrant or milder disease in one generation can cause a completely penetrant disease in the next (Campbell et al., 2014; Acuna-Hidalgo et al., 2015; Lauritsen et al., 2017; Wright et al., 2019b; Mastromoro et al., 2020).

Somatic mosaicism can also rescue an individual from disease, through cellular reversion that reduces the expressivity of a phenotype. For example, somatic reversions have been observed in several cell lineages from individuals with immunodeficiency caused by biallelic variants in *DOCK8*, including variants that correct or remove germline PTVs, and recombination events that attenuate or remove the deleterious variant from one allele. These somatic reversions improve overall survival time, but they are unable to completely eliminate the disease phenotype (Jing et al., 2014). Somatic reversion has been observed in other primary immunodeficiencies (Hou et al., 2021; Miyazawa and Wada, 2021) and may partially explain incomplete penetrance (Gruber and Bogunovic, 2020). Reversion of the clinical phenotype in individuals with recessive dystrophic epidermolysis (Pasmooij et al., 2010) and Fanconi anemia (Gross et al., 2002; Nicoletti et al., 2020) has also been identified. Remarkably, long-term remission from WHIM syndrome, caused by GoF variants in *CXCR4*, was seen in an adult who had undergone chromothripsis of chromosome 2 resulting in deletion of the disease allele in a single hematopoietic stem cell, leading to the repopulation of the bone marrow with the haploinsufficient CXCR4 cells (McDermott et al., 2015; Heusinkveld et al., 2017).

### Epigenetics

Epigenetic modifications are molecularly heritable changes that alter gene expression without altering the DNA sequence itself, including DNA methylation, histone modifications, and microRNA (miRNA) expression (Weinhold, 2006). Differential epigenetic modifications between individuals carrying the same pathogenic genotype can potentially account for incomplete penetrance and variable expressivity of the phenotype. DNA methylation is important in the control of tissue-specific gene expression, alternative splicing, prevention of cryptic initiation of transcription from alternative promoters, and X chromosome inactivation, all of which have been shown to affect the progression of disease (Velasco and Francastel, 2019). Studies of MZ twins that are discordant for disease phenotypes have highlighted how epigenetic mechanisms could affect the penetrance or expressivity of disease (Castillo-Fernandez et al., 2014). For example, MZ twins with neurofibromatosis, caused by variants in *NF1*, showed significant discordance in the presence of tumors and severity of scoliosis, suggesting that additional non-hereditary factors were modifying their phenotypes (Rieley et al., 2011). Similarly, one MZ twin with a pathogenic homozygous variant in *GBA* was diagnosed with Gaucher disease, while the other was clinically asymptomatic (Lachmann et al., 2004; Biegstraaten et al., 2011), and differences in their epigenome were posited as a mechanism to explain this discordance. However, epigenetic studies are generally more challenging than genetic studies as variation may be both tissue and time-specific, making it harder to elucidate how epigenetic mechanisms affect the penetrance of such genotypes. One suggested mechanism is that epigenetics may compensate for the presence of a deleterious variant, and segregate through several generations without any ill effects until the epigenetic modifications are no longer functional (Tolmacheva et al., 2020). This has been seen in Xq24 microdeletions that are inherited from mothers with extremely skewed X-chromosome inactivation, which modifies the penetrance (Tolmacheva et al., 2020). Skewed X inactivation is also suggested to be a cause behind the clinical heterogeneity in Klinefelter syndrome (Skakkebaek et al., 2020). Epigenetic mechanisms have also been suggested to partially compensate for deletions in healthy carriers of *IMMP2L* deletions, which cause ID and NDD, as reduced DNA methylation levels were seen in healthy carriers but not in affected offspring (Vasilyev et al., 2021).

Another mechanism by which epigenetic changes may affect the penetrance of monogenic diseases is *via* miRNAs, small non-coding RNAs that regulate the gene expression (Catalanotto et al., 2016). One miRNA can influence multiple genes, and a gene can be affected by several miRNAs, potentially highlighting how variants in one miRNA may lead to multiple downstream phenotypic effects (Wallace et al., 2020). Differential miRNA expressions can be caused by genetic variation, and variants within miRNA could, thus, affect the allelic expression and modify the penetrance or expressivity of monogenic diseases (Cammaerts et al., 2015). The expression of numerous miRNAs may affect the penetrance and expressivity in hereditary breast and ovarian cancer (HBOC) (Tommasi et al., 2021); incomplete and age-dependent penetrance is common in carriers of pathogenic variants in *BRCA1* and *BRCA2,* and variation in several miRNAs that bind the 3′UTRs and downregulate the expression of both genes have been linked with an increased risk of earlier onset cancer (Chen and Parmigiani, 2007; Chang et al., 2011; Moskwa et al., 2011; Sun et al., 2013; Tommasi et al., 2021).

## Global Modifiers

### Threshold Model of Disease

**FIGURE 4:**
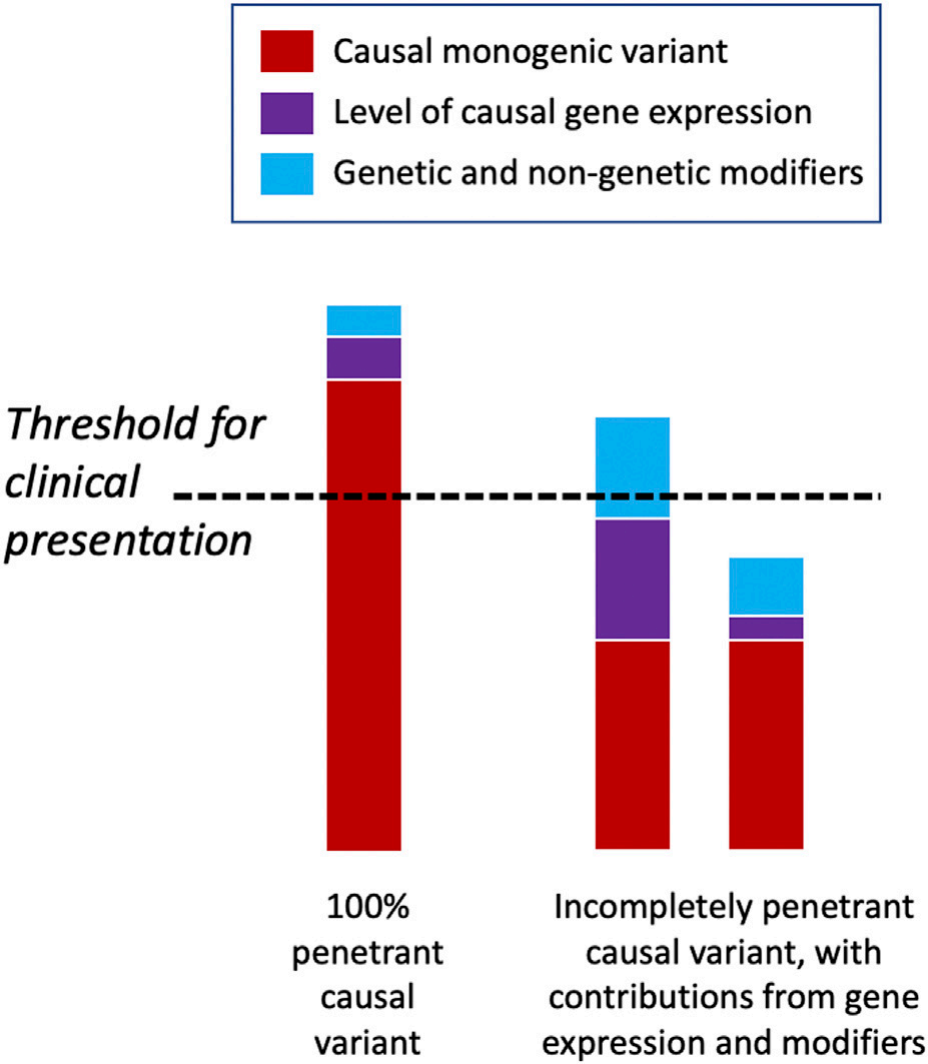
Threshold model of disease. Some deleterious monogenic variants are sufficient to cause the disease alone and do not need any genetic modifiers to cause the disease phenotype. Other monogenic variants may be incompletely penetrant and only display a disease phenotype when accompanied by other genetic or non-genetic factors that raise them above the clinical threshold for disease presentation. In the latter scenario, individuals may have the same underlying causal variant but have very different phenotypic presentations depending upon their modifying factors.

There may be a threshold that has to be met for the manifestation of a clinical disease phenotype, and genetic and other factors may vary in their relative contribution to meeting this threshold for different diseases and in different individuals (Figure 4) (Walsh et al., 2020). Some highly penetrant monogenic disease variants may always be sufficient to push the genetic burden above the threshold of the disease, although secondary variants may still contribute to severity (Pizzo et al., 2019). For example, Dravet syndrome (DS) is a highly penetrant and devastating form of childhood epilepsy caused by *de novo* LoF variants in *SCN1A* (Ding et al., 2021). Although DS displays considerable clinical heterogeneity within families and severity may relate to background genetic variation (Hammer et al., 2017), there are no known modifiers that protect against the effects of the primary causal variant; the LoF variant alone is sufficient to push the individual above the threshold for disease and other variants can only change the severity of the phenotype above this point. Individuals with monogenic variants that are causative of disease alone and, thus, are already above the threshold for disease can be further modulated by secondary monogenic variants in related genes that also cause the same phenotype, and the accumulation of these PTVs is associated with a more severe phenotype as the burden is pushed way beyond the threshold (Bertolini et al., 2020). For example, in monogenic polycystic kidney disease, individuals with PTVs in each of the causative genes, *PKD1* and *PKD2*, present with a much more severe disease than those with just one PTV (Arora et al., 2020). Many monogenic disease-causing variants have been found to have secondary genes or loci that affect the severity of their related clinical phenotypes (Posey et al., 2017; Pizzo et al., 2019) (Table 3).

**TABLE 3:** Examples of monogenic conditions affected by a putative second genetic locus that modifies the phenotypic expression.

Disease	Causal gene	Modifier gene/locus	Phenotypic effect
Cystic fibrosis	*CFTR*	*TGFB1* (Racanelli et al., 2018)	Increased severity of lung disease
		*IFRD1* (Gu et al., 2009)	Earlier age of the onset of chronic infection
		*DCTN4* (Emond et al., 2012; Viel et al., 2016)	
Sickle cell disease	*HBB*	*BCL11A* (Bae et al., 2012)	Prolonged production of fetal hemoglobin and reduced disease severity
		*HBS1L-MYB* (Bae et al., 2012; Steinberg and Sebastiani, 2012; Chang et al., 2018; Allard et al., 2021)	Decrease in disease severity
		*CLCN6* (Wonkam et al., 2020)	
		*OGHDL* (Wonkam et al., 2020)	
Long QT syndrome	*KCNQ1*	*NOS1AP* (Crotti et al., 2009)	Modulate the risk of arrythmias
	*KCHN2*		
	*SCN5A*		
X-linked retinitis pigmentosa	*RPGR*	*IQCB1* (Fahim et al., 2012)	Increase in disease severity
		*RPGRIP1L* (Khanna et al., 2009)	
		*CEP290* (Kammenga, 2017)	
Bardet-Biedl syndrome	*BBS10*	*CCDC28B* (Cardenas-Rodriguez et al., 2013)	Increase in disease severity
Spinal muscular atrophy	*SMN1*	*PLS3* (Oprea et al., 2008)	Reduction in disease severity
		*SNM2* (Calucho et al., 2018)	
Fragile X syndrome	*FMR1*	*COMT* (Crawford et al., 2021)	Reduction in disease severity
Spinocerebellar ataxia 17	*TBP*	*STUB1* (Magri et al., 2022)	Changes from non-penetrant to penetrant
Phenylketonuria	*PKU*	*SHANK* gene family (Klaassen et al., 2021)	Protective effect on cognitive development in untreated patients

In contrast, some monogenic disease-causing variants may be partially tolerated and transmitted through unaffected generations unnoticed, until they surpass the threshold for causing disease in the presence of other contributory factors. For example, large copy number variants (CNVs) are well-known causes of NDDs, but some—such as recurrent 16p12.1 deletions (Hanson et al., 2015)—have been widely observed to be inherited from unaffected parents. In this case, the penetrance of a phenotype that is severe enough to present clinically requires an additional variant that modulates the primary genetic variant (Servetti et al., 2021) supporting a “two-hit” model of NDDs (Girirajan et al., 2010). Similarly, deleterious variants in *CNTNAP2* and *LRRC4C* are insufficient to cause the disease alone but together may impair the development and function of synapses (Maussion et al., 2017; Um and Ko, 2017), suggesting a possible digenic mechanism for modulation of phenotypes (Poot, 2015). In many cases, however, there are likely to be numerous factors that affect whether an individual lies above or below the disease threshold, including the overall deleteriousness of the primary causal variant(s), the level of expression of the causal gene or isoform, and other genetic and non-genetic modifiers (Figure 4). Global modifiers that might affect penetrance and expressivity include polygenic risk, genetic compensation, variation in the NMD efficiency, family history, age, sex, and environmental factors.

### Polygenic Risk

The penetrance and expressivity of genotypes can be altered through the accumulated impact of many common genetic variants throughout the genome. The “omnigenic” model proposes that due to their interconnected nature, variants in gene-regulatory networks that are expressed in disease-relevant cells or tissues may affect the functioning of “core” disease-related genes due to effects on genes outside of the core pathways (Boyle et al., 2017), suggesting that many unrelated variants contribute to the presentation of a phenotype. Proposed as a factor in the inheritance of complex traits, this polygenic architecture could potentially also affect the presentation of monogenic conditions in a similar way, through non-coding variation that affects overall gene regulation, and many loci have been shown to additively affect expressivity and penetrance of monogenic variants in model organisms (Schell et al., 2022).

Genome-wide association studies (GWAS) have uncovered thousands of susceptibility loci for hundreds of diseases (Buniello et al., 2019), suggesting that the polygenic background can either predispose (Fahed et al., 2020) or protect individuals from diseases (Chami et al., 2020). Polygenic background can be quantified into a polygenic risk score (PRS) (Oetjens et al., 2019; Lewis and Vassos, 2020) and potentially used as a tool for the prediction of the overall disease risk in both monogenic and polygenic disorders (Khera et al., 2018). PRS associations highlight the additional risk of polygenic components in affecting the severity of monogenic disease, with the polygenic risk being shared across monogenic variant carriers and the general population (Kuchenbaecker et al., 2017). The effect of PRS has been widely explored to improve clinical interpretation of the penetrance of pathogenic variants across a range of monogenic conditions, including numerous familial cancer syndromes (Huyghe et al., 2019). The penetrance estimates for individuals with a pathogenic *BRCA1* or *BRCA2* variant range from 45 to 85% for breast cancer and from 10 to 65% for ovarian cancer (Petrucelli et al., 1993; van der Kolk et al., 2010), some of which can be explained by a polygenic background (Kuchenbaecker et al., 2017; Lee et al., 2019; Gallagher et al., 2020). Using a PRS generated from breast cancer GWAS, it has been shown that individual carriers of monogenic variants have risk differences of over 10% between the top and bottom PRS deciles (Kuchenbaecker et al., 2017). Interestingly, the majority of the SNPs identified as polygenic risk variants in breast cancer are common non-coding variants within regulatory regions, the target genes of which overlap with other known somatic cancer driver genes (Michailidou et al., 2017). Polygenic risk can also have a large effect on phenotypic diversity, even within individuals who have a known monogenic variant, illustrating that the genetic architecture for many diseases can be viewed as a spectrum rather than a binary classification of clinically symptomatic versus asymptomatic (Walsh et al., 2020). Although the overall polygenic contribution to the disease phenotype can be weaker in individuals with a monogenic variant (Harper et al., 2021), it can be useful in predicting overall penetrance and risk stratification.

### Genetic Compensation

The phenomenon of genetic compensation (or genetic buffering), where another gene or genes in a network can functionally compensate for LoF variants, has been shown in model organisms (Leopold et al., 2021) and hypothesized to play a role in incomplete penetrance in humans (Buglo et al., 2020). The upregulation of related genes or pathways or the differential expression of compensating alleles can help suppress a disease phenotype (Jordan et al., 2015), either through a small number of compensatory mechanisms or *via* a global shift in the gene expression. The functional redundancy of genes and rewiring of affected genetic networks may affect the penetrance and expressivity of corresponding phenotypes, and the consequence of a pathogenic variant may be influenced by variation across the genome (Payne and Wagner, 2015) and explain why certain LoF variants are tolerated by some individuals but not others (Subaran et al., 2015; Sulem et al., 2015). Haploinsufficiency can influence the expression of other genes in the same network, to maintain homeostasis or suppression of disease phenotypes (El-Brolosy and Stainier, 2017). The functional loss of one gene can be compensated for through functional redundancy (Chen et al., 2013). Genes that contain high numbers of PTVs in general population cohorts and thus are less likely to cause adverse phenotypes were found to belong to larger gene families than genes that contain known pathogenic PTVs (Ng et al., 2008), suggesting functional redundancy as a mechanism affecting penetrance (Hunter, 2022). Further research is needed to find robust evidence of this mechanism in humans.

### Nonsense-Mediated Decay Efficiency

The efficiency of NMD varies between individuals (Huang and Wilkinson, 2012), which could act as a potential modifier of penetrance and expressivity of PTVs targeted by NMD, irrespective of the specific causal variant(s) (Sarri et al., 2017). The variation in the NMD efficiency across codons, genes, cells, and tissues can affect disease pathology (Miller and Pearce, 2014; Sarkar et al., 2019; Sato and Singer, 2021). In studies of model organisms, the variant alleles that caused milder phenotypes were those that exhibited more NMD, with reduction in NMD being correlated with a more severe phenotype (El-Brolosy and Stainier, 2017). In this case, NMD could either help trigger a compensatory response, or haploinsufficiency could produce a milder phenotype than accumulation of truncated proteins. Variants in genes that encode the NMD machinery, or that either downregulate or remove NMD activity, have been linked to several NDD and ID syndromes, including variants in *UPF2* (Hildebrand et al., 2020), *UPF3A* (Nguyen et al., 2012), *EIF4A3* (Miller et al., 2017), *SMG8* (Alzahrani et al., 2020), and *RNPS1* (Nguyen et al., 2013), highlighting its importance in development and phenotypic expression. Common polymorphisms within the NMD pathway have been suggested to cause differences in NMD efficiency (Khajavi et al., 2006; Dyle et al., 2020), which could help explain differences in the expressivity of diseases caused by haploinsufficiency, with severity linked to whether they trigger NMD or not. Interindividual variability in NMD efficiency has the ability to alter the expressivity of genetic variants, by converting the cause of the disease phenotype from dominant-negative to haploinsufficiency, or vice versa (Supek et al., 2021). For example, two patients with the same PTV in the *DMD* gene displayed different clinical phenotypes, with one diagnosed with Duchenne muscular dystrophy, and the other with the milder Becker muscular dystrophy; here, the difference in the phenotype was suspected to be caused by weaker NMD efficiency in the less severely affected patient, which resulted in the production of the damaged but still partially functional DMD protein (Kerr et al., 2001; Torella et al., 2020).

### Family History

Family history can be seen as a crude but effective proxy for the combined effect of many shared genetic and environmental modifiers of disease phenotypes. In many cases, the pathogenicity and penetrance of variants in monogenic diseases have only been determined through studies of large families with multiple affected individuals, which can make it difficult to disentangle the relative contribution of different modifiers. Family history is a well-known major risk factor for hereditary cancer syndromes, and the number of affected relatives increases the risk of a pathogenic variant carrier developing cancer (Brewer et al., 2017). Although the evidence base for estimating penetrance in individuals without a family history is currently very limited (Turner and Jackson, 2020), individuals identified with a pathogenic variant for a heritable monogenic disease but without a family history of that disease may have a lower penetrance than those with a family history (Moreno-De-Luca et al., 2015; Wright et al., 2019a, Jackson et al., 2022).

Evaluating genetic differences between affected and unaffected carriers in the same family—such as *de novo* variants or unique combinations of modifiers—can be informative for understanding penetrance. It has been shown that children with monogenic NDDs have an excess of other damaging genetic variants compared to their either mildly clinically affected or asymptomatic carrier parents, with the extra genetic burden being enriched in genes that are highly expressed within the brain and in neurodevelopmental pathways (Pizzo et al., 2019). Similarly, children with 22q11.2 deletion syndrome display a wide variability in IQ scores that is highly correlated with the scores of their immediate relatives (Olszewski et al., 2014). The IQ of individuals affected by 22q11.2 deletion syndrome follows a normal distribution curve, similar to that of the general population, only 30 points lower (De Smedt et al., 2007). The significant association seen between parental and proband IQ (Klaassen et al., 2016; Davies et al., 2020) suggests that inherited genetic variants associated with intelligence may alleviate some of the deleterious impact of the 22q11.2 deletion on phenotypic presentation. The heritability of intelligence may be driven either by the cumulative effect of many common small-effect variants, similar to the heritability within population cohorts (Davies et al., 2011), or by a small number of rare high-effect variants. Similarly, individuals carrying 16p11.2 deletions present with variable phenotypic diversities (Moreno-De-Luca et al., 2015; Fetit et al., 2020) and are frequently present in “healthy” general population cohorts (Rosenfeld et al., 2013), albeit with a range of cognitive and neuropsychiatric difficulties despite none of them reaching traditional clinical diagnosis threshold levels (Stefansson et al., 2014). Within these carrier individuals, the best overall predictor of the phenotype was that of the average of their parental phenotype for the traits of interest, with individuals displaying deleterious effects relative to their phenotypic family background (Polyak et al., 2015; Evans and Uljarević, 2018).

### Age

It can be argued that penetrance is an almost meaningless concept without specifying an age threshold as many diseases do not present until later in life. As we age, gene expression and chromatin structure across the genome change, which can increase the penetrance or expressivity of disease (Brookes and Shi, 2014; Bashkeel et al., 2019). Expression of certain genes can cause change in a predictable way throughout life, with some only being expressed in the foetus or during early childhood, and others only after this developmental period. For example, the relative proportion of two protein subunits in the NMDA receptor alters with age due to the varying expression levels of the two genes, *GRIN2A* and *GRIN2B*, which can alter phenotypic expression of deleterious variants in these genes; prenatally expressed *GRIN2B* is linked with severe cognitive defects from birth, while postnatally expressed *GRIN2A* is linked with epilepsies in childhood and schizophrenia in adults (Strehlow et al., 2019). Studies of individuals who are below the age-penetrant threshold for known age-dependent diseases could explain why some pathogenic variants are found in apparently asymptomatic population cohorts. Classical examples of conditions where penetrance increases with age include cancer predisposition syndromes such as Li-Fraumeni (Correa, 2016), Lynch Syndrome (Biller et al., 2019), and HBOC (Chen and Parmigiani, 2007), where penetrance is affected by the accumulation of DNA damage over time (White et al., 2014). Meta-analysis studies have shown that the cumulative breast cancer risks for *BRCA1* and *BRCA2* pathogenic variant carriers by age 70 are 57–65% and 45–49%, respectively (Antoniou et al., 2003; Chen and Parmigiani, 2007), highlighting the difficulties with predicting the course of disease even in known pathogenic variant carriers and the importance of considering family history as well as other genetic and environmental factors (Lee et al., 2019). Age-dependent penetrance of cognitive phenotypes is also seen in diseases caused by the slow accumulation of aberrant proteins, where variation can affect the rate at which the protein accumulates (Chiti and Dobson, 2017). For example, retinitis pigmentosa (RP) has been suggested to be caused by retention of misfolded proteins, which leads to upregulation of genes that encode for proapoptotic machinery, and leads to apoptosis of photoreceptor cells, accumulating damage over time and eventually reaching the disease threshold and causing penetrant disease (Rose and Bhattacharya, 2016). Age-dependent penetrance may also be caused through gradual loss of neurons, causing the associated disease phenotype when the number of surviving cells drops below a certain threshold or overcomes brain plasticity (Magrinelli et al., 2021). For example, progressive and late occurring neurological manifestations in patients with *DNMT1* variants may originate from the gradual loss of DNA methylation over time, affecting adult neurogenesis (Velasco and Francastel, 2019).

The penetrance of age-dependent variants, present a diagnostic and prognostic challenge for individuals with such genotypes (Kalia et al., 2017). Previously, testing for many conditions early in life was not possible, and so little is known about long-term effects of mildly deleterious variants. Variants in *HFE* cause hereditary hemochromatosis, which can lead to iron overload in adulthood, and were previously thought to be an adult-onset condition. However, healthy cohort studies of children have shown that the effects of homozygous variants in *HFE* can be seen in childhood and that the cumulative effect of excess iron over a lifetime may affect the penetrance of numerous iron-related diseases (Kim and Connor, 2020). Recent population studies of adults have also shown substantially higher morbidity in homozygous *HFE* variant carriers with increasing age (Pilling et al., 2019). In this case early identification of individuals at risk can help with monitoring disease progression and introducing timely interventions (such as blood donation).

### Sex

Sex can affect the penetrance and expressivity of some genetic disorders, most obviously when deleterious genetic variants occur on the X chromosome, with hemizygous males more phenotypically affected than heterozygous females. Although differences in the penetrance of inherited variants based on sex have been reported in a variety of disorders (Cooper et al., 2013), mechanisms behind sex-dependent penetrance outside those that occur on the X chromosome are mostly unknown. However, there are widespread sex-biased differences in gene expression (Oliva et al., 2020), so differences in penetrance of phenotypes are also likely to be common. Females are less likely to be diagnosed with neurodevelopmental disorders than males, with a fourfold increase in the number of males diagnosed with autism spectrum disorders (ASD) compared to females (Scott et al., 2002; Christensen et al., 2016), suggesting that there may be a female protective effect that affects the penetrance of such conditions (Jacquemont et al., 2014). Girls diagnosed with ASD have an increased number of CNVs compared to boys with the same diagnosis, and asymptomatic mothers with children diagnosed with NDDs or ASD had a higher genetic burden of deleterious variants than fathers (Polyak et al., 2015), suggesting there may be some other cause for the incomplete penetrance and variable expressivity in females compared to males. However, females are ascertained at a closer frequency to males when they are more severely affected, suggesting some bias in clinical ascertainment due to differing phenotypic presentations between the sexes (Ratto et al., 2018), supported by the fact that males were more likely to be referred for genetic testing than females carrying the same autosomal variant (Russell et al., 2011).

### Environment

The environment can affect disease penetrance or expressivity in both a negative and positive manner and includes diet, drugs, alcohol intake, physical activity, ultraviolet light, *in utero* exposures, education, and socio-economic status, among many others factors. Epigenetic factors can provide a mechanistic link between the environment and gene expression (Dolinoy et al., 2007; Cavalli and Heard, 2019; Safi-Stibler and Gabory, 2020), and studies of the human microbiome can also explain some extreme variability in genotype–phenotype presentation (Sanna et al., 2019). However, although gene–environment interactions are likely to be widespread, they are often extremely hard to prove as the complete and systematic collection of an individual’s environment is almost impossible, and detailed relevant exposure data are rarely available alongside genetic data.

Inborn errors of metabolism perhaps provide the simplest examples of monogenic diseases where both a pathogenic genotype and an environmental exposure are required to cause disease (van Karnebeek and Stockler, 2012). A clear example of the dietary impact on phenotypic variation is phenylketonuria, a rare autosomal recessive disease that is usually detected through newborn screening, whereby individuals who have damaging biallelic variants in *PAH* can be put on a low phenylalanine diet to avoid serious disease progression (Flydal and Martinez, 2013; Al Hafid and Christodoulou, 2015). Later onset monogenic disease penetrance can also be affected by the environment, as seen in several cancer syndromes, including colorectal cancer, where inherited genetic variants interact with dietary variables and BMI to confer the overall risk (Lee et al., 2015). Cancer susceptibility can also be altered through gene–environment interactions such as smoking or sunburn, which can accelerate the accumulation of somatic variants that contribute toward tumorigenesis (Newcomb and Carbone, 1992; Wu et al., 2016). Similarly, environmental exposure to cigarette smoke, air pollution, and other airborne toxins can cause accumulation of unfolded or misfolded proteins and therefore affect the penetrance or expressivity of chronic lung disease (Wei et al., 2013). Individuals who carry a damaging monogenic variant may also be more susceptible to some environmental exposures, which can affect phenotypic severity (Tukker et al., 2021). For example, cystic fibrosis is characterized by progressive damage to the lungs, and non-genetic factors may account for up to 50% of the clinical variation seen (Collaco et al., 2010). Environmental factors such as smoking, air pollutants, temperature, and high-fat diets have all been shown to affect the severity and progression of disease (Collaco et al., 2010; Collaco et al., 2011; Schindler et al., 2015; Tukker et al., 2021), and the specific *CFTR* variant can also modulate how much environmental impact has on disease severity (Collaco and Cutting, 2008). Environmental factors can also affect the presentation of disease in primary atopic disorders, commonly seen as monogenic allergic disorders, where diet, microbiome at the epithelial-environment interface, presence/extent of infection, and psychological stress can all affect the penetrance or expressivity of the related phenotype (Sacco and Milner, 2019).

## Challenges Within Determining Penetrance and Expressivity

### Incomplete Penetrance Challenges Definitions of Pathogenicity

Determining the penetrance and expressivity of a variant can be difficult because it is sensitive to ascertainment context, and many studies are designed to enable the discovery of causative pathogenic variants in clinically affected individuals rather than to analyze effect sizes in populations (Manrai et al., 2016). This has been demonstrated in recent studies that stress the importance of cohort background for the determination of penetrance (Goodrich et al., 2021; Mirshahi et al., 2021). Investigating clinically classified pathogenic variants in large population cohorts can provide additional information about penetrance and expressivity (Kingdom et al., 2022), or determine whether variants or genes have been misclassified (Wright et al., 2019a). However, finding low penetrance pathogenic variants in large numbers of asymptomatic individuals challenges the concept of pathogenicity, particularly in the absence of known modifiers. What does it mean to describe a genotype as pathogenic if it is frequently found in individuals without disease and no explanation as to why? Reclassification of previously reported pathogenic variants occurs frequently, with variants first classified prior to the release of large population datasets showing a higher rate of reclassification (Harrison and Rehm, 2019). A study reappraising pathogenic variants in Brugada syndrome showed that only one gene (*SCN5A*) out of 21 could be definitively identified as causal (Hosseini et al., 2018), and another study has raised doubt over the involvement of 11/58 genes thought to cause inherited monogenic retinal disease (Hanany and Sharon, 2019). Variants that show low penetrance or a wide range of expressivity can also be potentially classified as risk alleles rather than causative variants. Some *CFTR* variants have been classified this way, with variations in cystic fibrosis phenotypes from very mild to very severe, and over 1900 different genotypes have been reported (Collaco and Cutting, 2008; Guillot et al., 2014; Pereira et al., 2019). Many genotype–phenotype associations are only reported once, or they are reported several times but with inconsistent results due to differences in data collection, differences in methods, or differences in cohort ascertainment. Associations can also differ due to poor annotation of coding genes, lack of relevant functional information for non-coding regions, sequencing and annotation errors, and varying penetrance and expressivity, making a simple binary classification of many genetic variants very difficult.

### Monogenic Versus Polygenic Disease

An overlapping genetic basis between complex traits and monogenic conditions is becoming increasingly apparent across the genome. Deleterious variants in genes causative of monogenic disease can be further dysregulated by non-coding variants that are associated with common traits, and monogenic forms of numerous common complex diseases have been identified (Peltonen et al., 2006; Chami et al., 2020; Hassanin et al., 2021). This overlap can cause considerable complexity when it comes to determining genotype–phenotype relationships (Freund et al., 2018). The prevalence of incomplete penetrance and variable expressivity raises questions as to what constitutes a disease state as opposed to extremes of normal phenotypic variation, especially within conditions that show significant clinical heterogeneity (Moreno-De-Luca et al., 2015), with many traits that constitute a clinical phenotype being the extreme end of either side of the bell curve of continuous distribution in the general population. Therefore, defining the penetrance of a genotype can be difficult, especially when there is ambiguity as to what defines the “disease state,” particularly for disorders where clinical features are only identified when they reach above a certain threshold (Senol-Cosar et al., 2019).

### Genetic Modifiers Are Hard to Identify

Relatively few studies have investigated low penetrant rare variants in detail or identified why such variants cause disease in one individual and not another. Despite increasing numbers of sequenced individuals, identification of genetic modifiers for monogenic conditions remains challenging. By definition, carriers of rare variants that cause monogenic conditions will be rare, with even fewer individuals having identical genetic modifiers that explain incomplete penetrance or variable expression. NGS approaches involving bioinformatic algorithms, including pathogenicity score-based prioritizations, can produce conflicting results and often need manual curation to identify candidate variants. A computational approach that could comprehensively analyze and prioritize candidate variants and potential modifiers would be a great advantage. Even in large population cohorts genome-wide analysis of genetic interactions lacks statistical power and can be easily affected by confounders (Wei et al., 2014). Many genetic modifiers are likely to be located in non-coding regions, making it challenging to determine their direct functional effect on the gene expression, especially as much of the genome is found to be bound by at least one transcription factor, many of which have no known function yet (The ENCODE Project Consortium, 2012). Improved computational approaches to identify candidate modifier gene interactions across the genome are needed (Lee et al., 2010), as well as identification of functional non-coding regions and the genes that they affect (Petrovski et al., 2015), and machine learning approaches such as DeepSEA and Enformer (Avsec et al., 2021) could improve annotation of these regions (Zhou and Troyanskaya, 2015).

## Future Perspectives

### Estimating Penetrance in Diverse Cohorts

Participants in population studies are usually investigated in a research-based environment rather than a clinical context, and despite rigorous phenotypic collection in some population studies, individuals involved may have subclinical manifestations of disease phenotypes that were unnoticed at the time of recruitment, or were not recorded in their medical histories (van Rooij et al., 2020). Lack of comprehensive phenotypic data can make using population cohorts to calculate the penetrance of genotypes very difficult but can at least provide a lower boundary of penetrance, with small clinical studies providing the upper boundary (Elfatih et al., 2021). Variant interpretation guidelines suggest that the penetrance of pathogenic variants in general population cohorts should be taken into account when calculating the overall penetrance of such variants (Kalia et al., 2017); however, even within healthy population cohorts there have been individuals identified with the associated phenotype but who have previously been described as unaffected (Chen et al., 2016), as well as individuals who display symptoms but are below the clinical threshold for classification. This is further complicated by conditions that are late-onset. In addition, genetic studies of human disease currently fail to capture the diversity that exists across the world, with most studies involving individuals of European descent (Lawson et al., 2020). This issue directly affects penetrance estimates, particularly as GWAS results and PRS may not be transferrable across diverse populations due to differing allele frequencies (Sirugo et al., 2019). Many deleterious variants may not be sufficient alone to cause disease, and therefore, estimates of penetrance need to consider the presence of other genetic variants and potential environmental effects (Figure 4). Calculating the etiological fraction of rare variants in specific conditions may provide a useful way to evaluate the probability that a variant detected in an individual with disease is causative (Walsh et al., 2017; Walsh et al., 2019), and disease-specific variant classifiers may also be of use (Zhang et al., 2021).

### Screening of Unselected Populations

As WGS becomes more common, individuals at risk of genetic disease will be identified earlier in life, potentially even from birth (Holm et al., 2018) and often prior to the appearance of relevant phenotypes. This can have a positive impact on overall health, with individuals who have no family history but a previously unknown high risk of disease being identified, enabling preventative screening or early treatment interventions. However, it can also cause harm through overdiagnosis; As seen across a number of population cohort studies, healthy individuals can harbor many potentially deleterious variants without ever developing any clinical symptoms. The effective use of genomic data requires a comprehensive understanding of functional genotype–phenotype correlations, which goes beyond that of Mendelian inheritance patterns. The increased sequencing of unselected populations, linked with electronic health records or other longitudinal phenotypic data, gives us an unprecedented ability to identify and reclassify rare variants and calculate penetrance estimates for a wide range of diseases and genotypes. These large-scale studies are crucial to inform the development of genomic screening programs (Holm et al., 2018; Wojcik et al., 2021) and the management of incidental or secondary findings. Discovery estimates of secondary findings vary from 1–3% of the population, with the majority of identified variants being those that confer susceptibility to cancer (Hart et al., 2019; Gordon et al., 2020). Incidental findings are predicted to be detectable at an appreciable level in individuals in the general population, many of whom may never develop the corresponding disease, suggesting that more robust determinations of pathogenicity are needed, including penetrance estimates for those without a family history of the disease (Johnston et al., 2012).

## Conclusion

Incomplete penetrance and variable expressivity are a significant concern for the correct interpretation of genetic variation and of diagnosing genetic disease. Correctly estimating penetrance and expressivity is challenging, with clinical cohorts and population studies both offering a different insight into its quantification. Although many monogenic disease-causing variants are fully penetrant, many are not, and improving our knowledge will involve WGS of population cohorts of increasing size and diversity, as well as functional studies of individual patients with specific clinical phenotypes. Achieving a mechanistic understanding of how incomplete penetrance and variable expressivity occur will help inform diagnostic and prognostic testing, clinical management, and accurate genetic counseling. To improve diagnostics and clinical interpretation of incompletely penetrant genotypes, a more sophisticated approach to disease genetics may be needed that integrates disease mechanism and specific variants with variation in levels of gene and tissue-specific isoform expressions and other genetic and non-genetic modifiers. Improving our knowledge of how variants exert their effects on genes, cellular pathways, and overall phenotypes will improve our understanding of disease and facilitate the development of new therapeutic interventions.
